# Perceived impact of heat stress on health and productivity of tropical female garment workers– a comparison between cool and hot months

**DOI:** 10.1186/s12889-025-22787-0

**Published:** 2025-04-25

**Authors:** Vabotra Chea, Sarin Chan, Natalia Borzino, Min Sze Pearl Tan, Jason Kai Wei Lee, Kinnaleth Vongchanh

**Affiliations:** 1 Faculty of Electrical Engineering, Institute of Technology of Cambodia, Russian Federation Blvd, P.O. Box 86, Phnom Penh, Cambodia; 2 Research and Innovation Center, Institute of Technology of Cambodia, Russian Federation Blvd, P.O. Box 86, Phnom Penh, Cambodia; 3 Singapore-ETH Centre, 1 Create Way, CREATE Tower, #06-01, Singapore, 138602 Singapore; 4 Campus for Research Excellence and Technological Enterprise (CREATE), National University of Singapore, Singapore, 138602 Singapore; 5 Human Potential Translational Research Programme, Yong Loo Lin School of Medicine, National University of Singapore, 10 Medical Drive, MD11, #03-12, Singapore, 117597 Singapore; 6 Department of Physiology, Yong Loo Lin School of Medicine, National University of Singapore, 2 Medical Drive, MD9, Singapore, 117593 Singapore; 7 Heat Resilience and Performance Centre, Yong Loo Lin School of Medicine, National University of Singapore, 27 Medical Drive, #03-01, Singapore, 117510 Singapore

**Keywords:** Occupational exposure, Hot temperature, Productivity loss, Heat stress disorders, Heat adaptation measures, Women working, Tropical climate

## Abstract

**Background:**

Challenging work conditions, characterized by high temperatures and humidity without the availability of adequate cooling systems, can put garment workers at an increased risk of heat stress. We examined the impact of heat stress on the health and productivity of young female garment workers, and the heat relief measures they took.

**Methods:**

We surveyed and compared a total of 753 female responses across three factories in tropical Phnom Penh, Cambodia, between the cool (November 2021 to January 2022, WBGT 25.2 ± 2.0^o^C) and hot months (April to June 2022, WBGT 29.0 ± 0.8^o^C). The surveys assessed perceptions of thermal comfort in the workplace, the effects of heat stress on heat-related symptoms and productivity, as well as the heat relief measures taken. Non-parametric tests were used to assess differences in responses between the cool and hot months.

**Results:**

During hot months, respondents reported an increase in heat-related symptoms (68% in cool months vs. 88% in hot months). Common symptoms included thirst (50% vs. 81%, *p* < 0.001), feeling hot (28% vs. 68%, *p* < 0.001), and heavy sweating (31% vs. 61%, *p* < 0.001). The perceived impact on productivity was greater during hot months (59% vs. 68%). Respondents perceived heat degraded their motivation (50% vs. 72%, *p* < 0.001), task speed (42% vs. 66%, *p* < 0.001), ability to do physical work (37% vs. 56%, *p* < 0.001), and understanding of tasks (18% vs. 31%, *p* < 0.001). Increasing water intake was the most common heat relief measure (87% vs. 95%, *p* < 0.001), while other strategies such as resting in front of a fan (32% vs. 36%) or pouring water over their head (20% vs. 21%) were similar between the cool and hot months (*p* > 0.05).

**Conclusions:**

Even a small increase in temperature could compromise workers’ health and work productivity. Workers had to seek heat relief measures all year round due to constant exposure to high temperatures and humidity. In face of a warming world, it is therefore pertinent that these heat-induced impacts are addressed to safeguard workers’ lives and livelihoods, and to ensure productivity in factories.

**Supplementary Information:**

The online version contains supplementary material available at 10.1186/s12889-025-22787-0.

## Background

Climate change presents numerous challenges to human well-being [1], particularly through an increase in heat stress [2]. Heat stress occurs when the body accumulates more heat than it can dissipate [3], leading to detrimental physiological effects [3–6]. It results from a combination of environmental factors (air temperature, air velocity, humidity, and solar radiation), metabolic heat from physical activities, and the type of clothing worn [3–6].

Recognized as an occupational hazard [7], heat stress affects workers in various industries [8], and its adverse effects on health and productivity are well documented [4]. Excessive heat exposure can result in physiological and psychological strains [5, 8], leading to heat-related symptoms and illnesses, dehydration, kidney problems, and cardiovascular complications [3, 4, 9, 10]. Moreover, occupational heat stress can diminish workers’ motivation and productivity, while increasing the risk of accidents [11].

The garment, footwear, and travel goods (GFT) sector is one of the sectors vulnerable to heat stress [12, 13]. This sector plays a vital role in Cambodia’s economy, with over 855,000 workers employed in 2023, over 80% of whom are women [14]. These workers often endure high temperatures and humidity, often with inadequate cooling systems in place [12, 13]. Heat generated from machineries and inefficient lighting may further elevate workplace temperatures [15]. Due to high energy costs, air conditioning is limited, with factories often relying on evaporative cooling systems which are ineffective in hot and humid conditions [16]. As climate change intensifies, the vulnerability of these workers to heat stress becomes more pronounced. This highlights the need for a comprehensive understanding of the impact of heat stress on GFT workers, and the heat relief measures they often use. Most studies on heat stress involve both men and women, with few specifically examining female workers [17, 18]. Women may be more prone to heat intolerance and respond differently to heat compared with men [17, 18]. Understanding how heat affects female workers is crucial, especially in the GFT sector. Studies on Cambodian GFT workers’ perceptions of heat stress are limited and often based on small, one-time samples, without comparing the impacts between cool and hot months, thereby ignoring seasonal variations [12, 13]. Regular exposure to heat, whether seasonal or year-round, can elevate the risk of heat stress [19], and even minor temperature changes can affect health and work performance [20]. It is important to conduct studies with the same people across the different periods, and compare them to capture the actual differences in the impacts [21, 22].

The objective of this study was to examine the seasonal impacts of heat stress on the health and productivity of garment workers, as well as their resourcefulness in dealing with heat stress. Specifically, we aimed to determine whether perceived impacts during cool and hot months differed, and how these variations could have resulted in different coping behaviors and measures used by garment workers.

## Methods

### Survey design

We adapted the survey questions from the High Occupational Temperature Health and Productivity Suppression (HOTHAPS) questionnaire [21], Project HeatSafe^1^, and previous studies [9, 10]. The survey was tailored to garment workers and translated into the native Khmer language. The survey comprised six sections: demographic information, work type, heat exposure, perceived impact of heat stress on health and on productivity, and heat relief measures used. The first two sections gathered basic information such as the age, education, job types, and employment duration of the respondents. The third section focused on heat exposure at work, asking respondents to rate the thermal environment of their workplaces during the three months prior to the survey. Respondents rated their experiences using a seven-point scale, ranging from “very cold” to “very hot”. We also asked respondents about the type of clothing worn and their thermal comfort levels. In the next two sections, we asked respondents if they experienced any heat-related symptoms in the three months prior to the survey, and their perceptions of how heat had impacted their productivity. Respondents were given a list of potential heat-related symptoms and ways in which heat might have impacted their productivity. They then selected multiple options reflecting their experiences during the three months prior to the survey. The last section explored the heat relief measures they used to cope with heat both at work and outside of work. All questions were closed-ended. Details of questionnaire are given in the Supplementary Material.

### Study site and selection of participants

Our study focused on three garment factories in tropical Phnom Penh, where most garment factories in Cambodia were located. Respondents included female sewing machine operators, assistants, team leaders, and supervisors from the sewing department. We specifically chose the sewing department as it was the most important, complex, and labor-intensive process in a garment factory. We excluded respondents with pre-existing medical conditions such as diabetes and hypertension.

### Sampling and data collection

Students from the Thermal Laboratory of the Institute of Technology of Cambodia conducted one-on-one face-to-face interviews in Khmer. We aimed to capture experiences during the historically hottest (April-June) and coolest months (November-January), based on indoor wet bulb globe temperature (WBGT). This was determined from the HOTHAPS program [21], which captured historical data from 1981 to 2021. From HOTHAPS, the mean WBGT was 29.3^o^C during the hottest months of April to June, and 26.8^o^C during the coolest months from November to January, indicating a 2.5^o^C difference [21]. Surveys were conducted twice, in February and July 2022, on normal working days. Each interview took 20 to 30 min. Respondents received KHR 6,000 (approximately USD 1.5) as an incentive. Respondents were not informed about the incentive until they had completed the survey. The required sample size for each period was determined to be 196, based on a 50% population proportion, 95% confidence level, and 7% margin of error [23].

### Ethical considerations

Our protocol was approved by the Cambodian National Ethics Committee for Health Research (Reference No. 304NECHR). Factory management gave their approval to conduct the surveys on their premises. Prior to the interview, respondents received information about the study and signed informed consent forms. Their anonymity was protected, and they could decline to answer questions or withdraw at any time. Interviews were conducted without the presence of the management team, thus removing any possible influence of the employers.

### Environmental measurements

We installed data loggers (HOBO MX1101) in the sewing lines to record dry bulb temperature and relative humidity at 15-minute intervals from July 2022 to June 2023. Equation 1 [24] was used to estimate indoor WBGT.

1 $$ WBGT{\rm{ }} = {\rm{ }}0.67{T_{pwb}} + {\rm{ }}0.33{T_a} - {\rm{ }}0.048\log \left( {ws} \right)\left( {{T_a} - {\rm{ }}{T_{pwb}}} \right) $$

Where: T_a_ was the dry bulb temperature in ^o^C; ws was wind speed, which was fixed at 1 m per second (m/s); T_pwb_ was the psychrometric wet bulb temperature and was estimated by Eq. 2 [25].

2 $$ \begin{array}{l}1556{e_d} - 1.484{e_d}{T_{pwb}} - 1556{e_w} + 1.484{e_w}{T_{pwb}}\\+ 1010\left( {{T_a} - {T_{pwb}}} \right) = 0\end{array} $$

Where: e_d_ and e_w_ were the saturation vapor pressures at dew point temperature and wet bulb temperature, respectively, and was calculated by the following:

$$ {e_d} = 6.106\exp \left( {17.27{\rm{ }}{T_d}/\left( {237.3 + {T_d}} \right)} \right) $$

$$ {e_w} = 6.106\exp \left( {17.27{T_{pwb}}/\left( {237.3 + {T_{pwb}}} \right)} \right) $$

### Data analysis

Descriptive statistics, including mean, standard deviation and frequency, were used to describe the demographics of respondents during the cool and hot months. We then assessed and compared the perceived impact of heat stress during these periods. As the data distribution was assumed to be non-normal, the non-parametric Wilcoxon Mann-Whitney test was used to gauge differences within variables. All analyses were performed using R Statistical Software (version 4.1.3), using the ‘gtsummary’ package [26]. We also computed the difference between cool and hot months (hot months– cool months), 95% confidence intervals, and *p*-values. Statistical significance was ascertained by *p*-values being lower than 0.05.

## Results

### Environmental measurements

Environmental data during the cool months (November 2021 to January 2022) was not captured due to logistical issues. In its place, data collected from November 2022 to January 2023 was used as a proxy for the environmental conditions during the cool months.

Environmental data from November 2021 to January 2022 (actual profiling period for the cool months) was extracted from the HOTHAPS program [21], and compared with that from November 2022 to January 2023 (proxy for the survey period for cool months). This is presented in Supplementary Material Table 1. Through this, we ascertained that there were no differences in the environmental data during the cool months from November 2021 to January 2022, and November 2022 to January 2023 (*p* > 0.05). Hence, proxy data from November 2022 to January 2023 could be used to represent the environmental conditions during the actual profiling period for the cool months in our study.

In all, the hourly mean dry bulb temperature recorded within the factories was 27.8^o^C (± 2.3^o^C) and 31.0^o^C (± 1.7^o^C), with a mean relative humidity (RH) of 74% (± 13%) and 80% (± 14%) during the cool and hot months, respectively. The hourly mean WBGT was 25.2^o^C (± 2.0^o^C) during the cool months and 29.0^o^C (± 0.8^o^C) during the hot months.

### Sample description

There was a total of 800 responses to our survey, with 753 valid responses (380 for cool months, 373 for hot months) obtained (Table 1). We obtained 161 responses from the same respondents in both the cool and hot months (i.e., repeated measures), which accounted for 42% and 43% of respondents in the cool and hot months, respectively. Our respondents were young females, with the mean age for both samples at 33 (± 7) years. Sewing machine operators represented 91% of the total responses. Over one-third completed primary school (47% and 50% for cool and hot months), while 37% and 39% finished secondary school.

**Table 1:** Demographics of surveyed workers in both the cool and hot months

	Overall, *N* = 753^1^	Cool months, *N* = 380^1^	Hot months, *N* = 373^1^
**Age (years)**	33 (7)	33 (7)	33 (7)
**Designation**			
Sewing machine operator	91%	91%	91%
Assistant	4%	4%	3%
Team Leader	5%	4%	5%
Supervisor	0.3%	0.3%	0.3%
**Education**			
Illiterate	3%	3%	2%
Primary	48%	47%	50%
Secondary	38%	37%	39%
High School	11%	12%	9%

^1^ Mean (SD); %

### Perceived thermal comfort

**Fig. 1:**
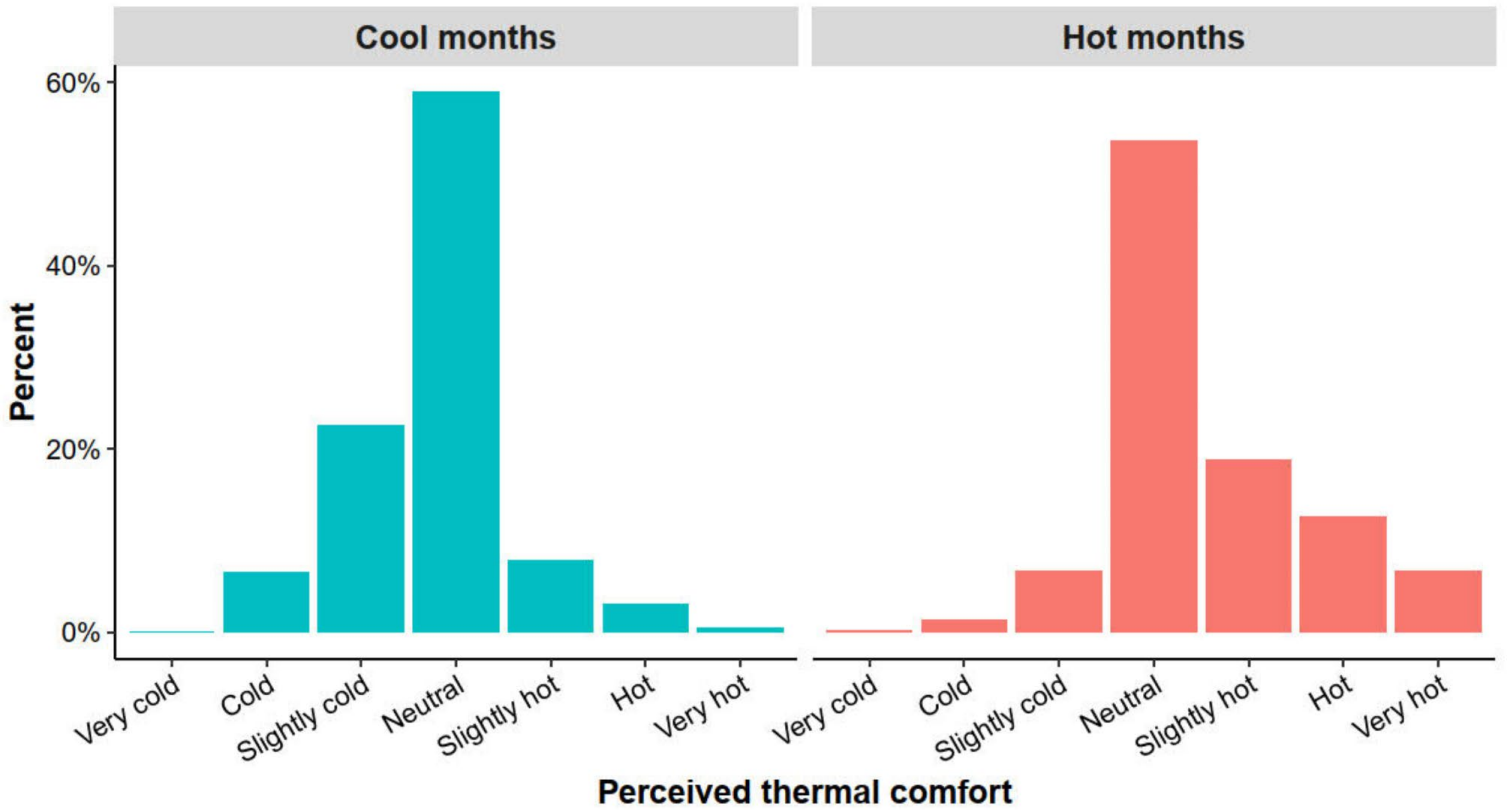
Perceived thermal comfort at the workplace during the cool and hot months

Figure 1 showed the perceived thermal comfort at the workplace for cool and hot months on a scale from “very cold” to “very hot”. Most workers felt “neutral” (59% vs. 54%). However, more workers perceived their thermal comfort as “slightly hot” (8% vs. 19%, *p* < 0.001), “hot” (3% vs. 13%, *p* < 0.001), and “very hot” (0.5% vs. 7%, *p* < 0.001) during the hot months.

### Choice of clothing worn

Over half of the workers preferred long sleeves (53% vs. 78%, *p* < 0.001), while others chose t-shirts (25% vs. 0%, *p* < 0.001) and short sleeves (21% vs. 22%) as their preferred working attire (Supplementary Material Table 2). Nearly all workers preferred long pants (99% for both periods). Additionally, more than half (56% vs. 54%) opted for two layers of outer clothing, including an additional uniform layer, with turtleneck t-shirts being common. More than half felt comfortable (57% vs. 75%, *p* < 0.001), while fewer felt moderately comfortable (41% vs. 24%, *p* < 0.001) in their chosen working attire.

### Impact of heat stress on heat-related symptoms

**Fig. 2:**
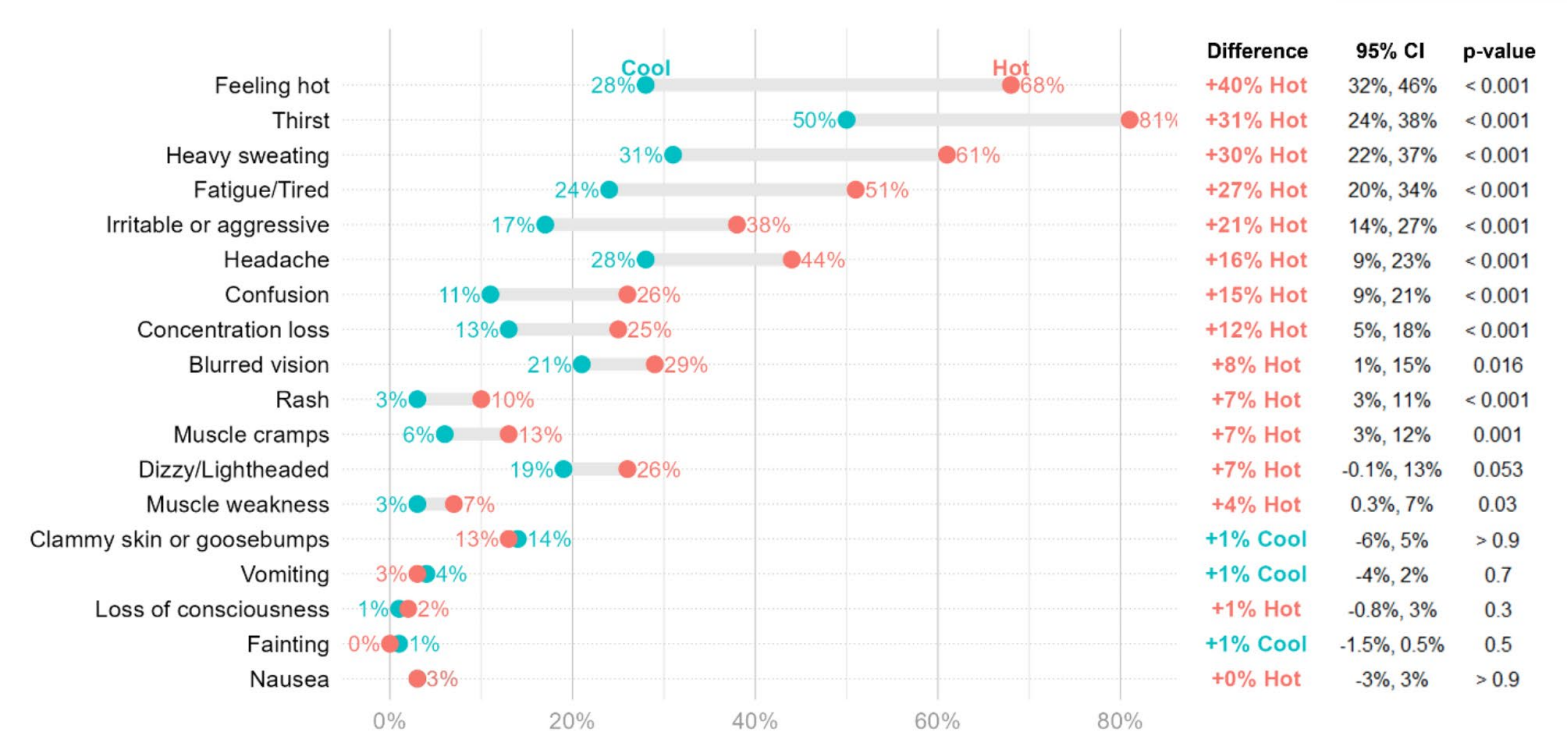
Respondents’ perceived heat-related symptoms during the cool and hot months

Respondents reported more heat-related symptoms during the hot months (88%) as compared to during the cool months (68%) (Fig. 2). Thirst was the most common symptom (50% in cool months and 81% in hot months, *p* < 0.001). Other symptoms like feeling hot (28% vs. 68%, *p* < 0.001) and heavy sweating (31% vs. 61%, *p* < 0.001) were also more prevalent during hot months, along with headache (28% vs. 44%, *p* < 0.001), irritability (17% vs. 38%, *p* < 0.001), confusion (11% vs. 26%, *p* < 0.001), concentration loss (13% vs. 25%, *p* < 0.001), rash (3% vs. 10%, *p* < 0.001), muscle cramps (6% vs. 13%, *p* = 0.001), and muscle weakness (3% vs. 7%, *p* = 0.03).

### Impact of heat stress on productivity

**Fig. 3:**
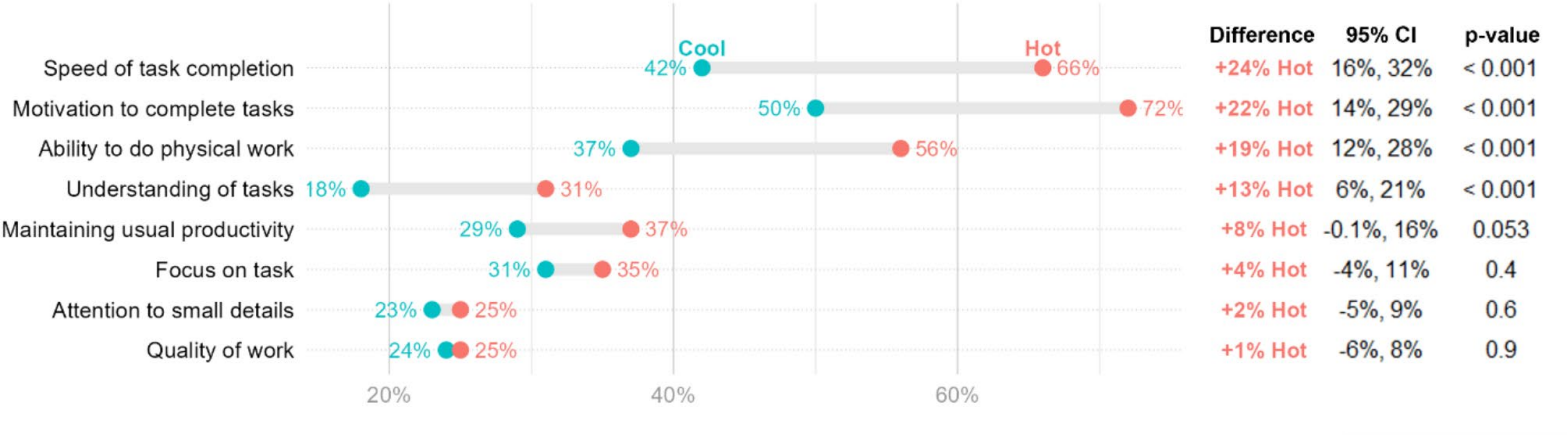
Respondents’ perceived impact of heat stress on productivity during the cool and hot months

In Fig. 3, respondents (59% during cool months and 68% during hot months) reported that heat degraded their ability to perform work in one or more aspects. This included reduced motivation to complete tasks (50% vs. 72%, *p* < 0.001), slower task completion (42% vs. 66%, *p* < 0.001), decreased ability to do physical work (37% vs. 56%, *p* < 0.001), and poorer understanding of tasks (18% vs. 31%, *p* < 0.001). Additionally, respondents (55% vs. 70%) indicated that heat impacted various aspects apart from their work performance. Respondents experienced an increase in feelings of tiredness (38% vs. 73%, *p* < 0.001) and sleepiness at work (29% vs. 45%, *p* < 0.001) (Supplementary Material Fig. 1). The respondents perceived a decrease in productivity (31% vs. 54%, *p* < 0.001), and reported taking more time to complete the same task (27% vs. 52%, *p* < 0.001).

### Heat relief measures

**Fig. 4:**
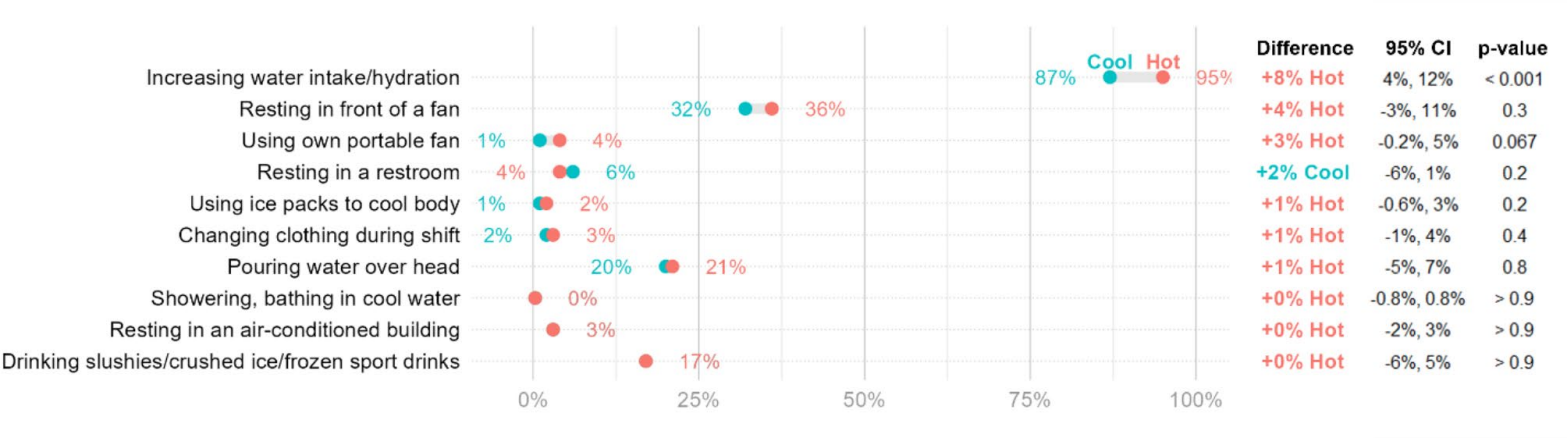
Respondents’ heat relief measures at work during the cool and hot months

Heat relief measures (Fig. 4) were largely consistent across two periods. The only difference was increasing water intake/dehydration in the hot months (87% vs. 95%, *p* < 0.001). In addition, we explored the measures used by respondents to cope with heat outside of their work, which is presented in Supplementary Material Fig. 2.

## Discussion

Our findings showed that more workers perceived their workplace thermal comfort as “slightly hot”, “hot”, and “very hot” during the hot months compared with the cool months. This is expected due to the common architectural designs of many Cambodian factories, which typically have brick walls and metal roofs that exacerbate external heat gain. As a result, workers often find themselves working in conditions of high temperatures and humidity [12, 13]. Our measurements indicated that hourly mean dry bulb temperature reached as high as 31.0^o^C (± 1.7^o^C) during the hot months, with a 3.2^o^C higher temperature between the cool and hot months. Relative humidity was also relatively high (74 ± 13% and 80 ± 14% for cool and hot months, respectively). The highest WBGT recorded during hot months was 32.1^o^C, with a mean of 29.0^o^C (± 0.8^o^C). Another study conducted in a garment factory in Phnom Penh has similarly reported a WBGT of 34.0^o^C during the hot months [12].

Respondents reported a higher number of several heat-related symptoms during the hot months. Our study confirmed that even small temperature increases could compromise the health of the workers exposed to extreme heat year-round. A study in India found similar trends, with 442 workers across 18 workplaces reporting that the perceived impact of heat stress was greater in hot months, with similar WBGT differences (1.8^o^C– 4.3^o^C) [20]. Additionally, a 1^o^C increase of the Universal Thermal Climate Index (UTCI) was associated with a 4% increase in reported symptoms [27].

High humidity levels in Cambodia can potentially hinder the body’s ability to dissipate heat via the evaporation of sweat [3, 6], resulting in heat-related symptoms such as feeling hotter, heavy sweating, thirst, fatigued/tiredness, and headache. In our study, 61% reported heavy sweating, 51% reported fatigue/tiredness, and 44% reported headache during the hot months. This is slightly higher than another study of 130 garment workers in Phnom Penh, with 45%, 34%, and 33% of respondents reporting heavy sweating, fatigue/tiredness, and headache, respectively [12]. Another study in Cambodia showed that a similar number of respondents experienced fatigue/tiredness (41%), but there was a higher number of headaches (93%) reported [13]. The differences between the findings may stem from the participant selection, as our study focused specifically on female workers in the sewing department, whereas others included male and female participants from other departments. In garment factories, workers are assigned to specific roles, such as sewing, ironing, packaging, with each demanding different levels of exertion. Men are often placed in more labor-intensive roles, particularly the ironing department where they face a greater exposure to heat.

Beyond physiological impacts, heat in the workplace can also affect worker productivity, as heat-related symptoms are strongly associated with worker performance [28]. Heat-induced fatigue and discomfort can impair physical performance, such as the speed of task completion and ability to do physical work, as well as cognitive abilities, including the ability to understand tasks. These were reported by a previous study [28], and by respondents in our study. More than half of our respondents reported having an impaired work performance during hot months (59% vs. 68%, *p* < 0.001). In contrast, another study found only 22% of respondents perceived their ability to work as being compromised by heat [13].

One-third of our respondents (32% vs. 33%) perceived heat to affect their job satisfaction, although this perception did not differ between two periods. Similar findings from a survey in India suggest that heat affects job performance but not job satisfaction [29]. In contrast, previous studies from Australia [28], Malaysia [30], and Iran [31] suggest that heat can influence job satisfaction, but factors like age, marital status, and skill level also play roles to influence this [30].

Interestingly, our respondents did not report any impact of heat stress on their focus on task, attention to detail, or work quality between both periods. It is unclear why these impacts were not different. One possible explanation is that workers had to maintain a high level of concentration on their tasks as they were employed via a piece-rate contract, with additional pressure exerted by supervisors to complete their tasks and the fear of losing their job if they did not perform up to expectations.

More than half of our respondents reported feeling less productive (54%) and taking more time to complete the same task (52%) during the hot months, which aligns with but exceeds the 41% and 14% respectively, as reported in a previous study [12]. Given the nature of the garment production’s assembly line, heat stress affecting individual workers could lead to productivity loss across the entire assembly line and ultimately the productivity of the entire factory.

Interestingly, the heat relief measures reported during the cool and hot months were similar. This suggests that workers were constantly exposed to high temperatures and sought heat relief measures year-round. Respondents used a variety of heat relief measures, with over 90% of them using ten different measures. Some strategies, such as resting in an air-conditioned building and changing clothing during their shift were rarely used. Access to air-conditioned areas was limited, as these spaces were typically reserved for offices and meetings. Additionally, our data revealed that more than half of the respondents preferred wearing 2–3 layers of outer clothing. From the interviews, respondents felt that wearing multiple layers could increase sweating, hence enhancing evaporative cooling.

87% of respondents increased their water intake to cope with the heat during the cool months, and this figure increased to 95% during the hot months (*p* < 0.001). This is higher than that reported in a previous study conducted in Cambodia (55%) [12]. It is important to note that while water intake was a widely used measure, it does not necessarily mean that the respondents were consuming a sufficient amount of water to ensure euhydration. Our study did not ask the respondents to specify the quantity of water they consumed, and this should be an area of focus for future studies. Water intake alone may not sufficiently reduce heat stress [32]. Factories could provide cool water or ice slurries, as these have been demonstrated to effectively reduce body core temperature [23]. The availability of cool water could also encourage workers to increase their water intake [10].

Additionally, implementing heat-related training programs could improve workers’ understanding of how they can take steps to prevent themselves from succumbing to heat-related illnesses [9]. Factories could also allow workers to take regular and longer breaks to recuperate, which could in turn enhance workers’ satisfaction, reduce turnover rate, and improve their overall productivity [10].

Similarly, the heat relief measures reported outside of work were similar across both the cool and hot months. Some measures such as resting in the shade and in front of a fan increased during hot months (*p* < 0.001). These findings align with the tendency of respondents to spend more time indoors when not working. Access to air conditioning was limited, with only 9% of respondents reporting resting in an air-conditioned room or home during both cool and hot months. Additionally, a previous study found that the amount of water consumed varied between seasons of the year [33]. Our study indicates that respondents drank more water before (*p* < 0.001) and during work (*p* < 0.001) on hotter days. This could be a result of increased sweating during the hot months.

### Limitations

This study has several limitations. First, our data relied on self-reported symptoms during the three months prior to the surveys, which could have introduced recall and self-reporting biases. Second, only female workers were included from the sewing department of three factories in Cambodia, which could have introduced selection bias. While it is adequate for drawing preliminary conclusions, this may not represent male workers or workers in other industries. Caution should be made when attempting to generalize the findings of this study.

## Conclusions

This study examined the perceived impact of heat stress on the health and productivity of 753 female garment workers in tropical Cambodia during the cool and hot months. Comparing data from both periods revealed there were greater impacts during hot months, confirming that even slight increases in temperature could compromise workers’ health and wellbeing. Symptoms like thirst, feeling hot, and heavy sweating were more prevalent in the hot months. Respondents perceived that heat also impaired their productivity, demonstrating a clear link between heat exposure and work productivity. Interestingly, the heat relief measures used by the respondents did not differ between the two periods, suggesting that workers experienced heat stress year-round and sought relief regardless of the season. These findings emphasize the need for implementation of heat management strategies in the GFT sector to safeguard workers’ lives and livelihoods, and to ensure workers’ productivity in face of a warming world.

## Electronic supplementary material

Below is the link to the electronic supplementary material.

Supplementary Material 1

## Data Availability

The datasets used and/or analysed during the current study are available from the corresponding author on reasonable request.

## Footnotes

1 Source: https://www.heatsafe.org/.
