# Supplementary material for: Perceived impact of heat stress on health and productivity of tropical female garment workers– a comparison between cool and hot months
Source: BMC Public Health. 2025 Apr 25;25:1543. doi: 10.1186/s12889-025-22787-0 (PMC12023358; doi:10.1186/s12889-025-22787-0)
Supplement: Supplementary file 1 — Supplementary Material 1 [file 12889_2025_22787_MOESM1_ESM.docx]

# Supplementary Materials

**Questionnaires for Garment Workers**

**Part 1: Concerning on demographics of interviewee**

| 1. Do you agree to participate in this survey? Remember, you can withdraw at any time. | ⃝ Yes  ⃝ No (Stop the survey) |
| --- | --- |
| 2. Name of the interviewee |  |
| 3. Age | years |
| 4. How tall are you? | m |
| 5. What is your approximate mass? | kg |
| 6. Gender | ⃝ Male  ⃝ Female |
| 7. Designation | ⃝ Worker  ⃝ Assistant  ⃝ Team leader  ⃝ Supervisor  ⃝ Other___________________________ |
| 8. Education | ⃝ Illiterate  ⃝ Primary  ⃝ Secondary  ⃝ High school  ⃝ University  ⃝ Other___________________________ |
| 9. Smoking | ⃝ Smoker  ⃝ Non-smoker  ⃝ Ex-smoker |
| 10. Consuming alcohol | ⃝ Yes  ⃝ No  ⃝ Ex-drinker |
| 11. Any existing illness? | ⃝ No  ⃝ Diabetes  ⃝ Hypertension  ⃝ Respiratory illness  ⃝ Other___________________________ |

**Part 2: Concerning type of the work**

| 1. Department | ⃝ Sewing  ⃝ Cutting  ⃝ Ironing  ⃝ Other__________________________ |
| --- | --- |
| 2. How long have you been employed here? | ___________________________years ___________________________months |
| 3. Have you been working here in different position?  If yes, what was your position? | ⃝ Yes  ⃝ No  Position: _________________________ |
| 4. How many hours per day do you usually work? | ___________________________hours  From____________ To_____________ |
| 5. Do you work near a direct heat source (e.g., naked flame/hot air/outdoors/radiant heat) | ⃝ Yes  ⃝ No |

**Part 3: Concerning on heat exposure at work**

| 1. Over the last 3 months, how do you judge the thermal environment in which you generally work? | ⃝ Very cold  ⃝ Cold  ⃝ Slightly cold  ⃝ Neither cold nor hot  ⃝ Slightly hot  ⃝ Hot  ⃝ Very hot |
| --- | --- |
| 2. What is your perception about outfit material? | ⃝ Comfortable  ⃝ Moderately comfortable  ⃝ Uncomfortable  ⃝ Other_________________________ |
| 3. Upper clothes | ⃝ Short sleeves  ⃝ Long sleeves  ⃝ Long-sleeves turtle neck  ⃝ Other_________________________ |
| 4. Lower clothes | ⃝ Short skirt  ⃝ Long skirt  ⃝ Long pants  ⃝ Other___________________________ |
| 5. How many outer layers (of clothes) do you wear? | ⃝ 1 layer  ⃝ 2 layers  ⃝ Thick cloth  ⃝ Other_________________________ |
| 6. (If Question 5 is thick cloth or more than one layer) Please specify reason. |  |
| 7. Do you feel hotter with outfit/uniform? Does the dress material increase heat stress? | ⃝ Sure  ⃝ Maybe  ⃝ Not sure  ⃝ No, not at all |

**Part 4: Concerning on impacts of heat stress on health**

| 1. Over the last 3 months, have you felt any of the following heat-related symptoms? | □ Feeling hot  □ Heavy sweating  □ Thirst  □ Fatigued/Tired  □ Headache  □ Irritable or Aggressive  □ Clammy skin or Goosebumps  □ Rash  □ Concentration loss  □ Muscle cramps  □ Muscle weakness  □ Vomiting  □ Nausea  □ Blurred vision  □ Dizzy/Lightheaded  □ Loss of consciousness  □ Fainting  □ Confusion  □ Convulsions/Seizures  □ Heart attack  □ Other_________________________ |
| --- | --- |

**Part 5: Concerning on impacts of heat stress on productivity**

| 1. Have you taken sick leave as a result of exposure to heat, or symptoms associated with heat stress in the last 3 months?  If yes, please specify approximately how many days. | ⃝ Yes  ⃝ No  _________________________hour/week |
| --- | --- |
| 2. During the last 3 months, have you been advised to take off due to heat-related sickness? | ⃝ Yes  ⃝ No |
| 3. During the last 3 months, have you visited a doctor due to heat-related sickness? | ⃝ Yes  ⃝ No |
| 4. During the last 3 months, have you been admitted in hospital/medical center due to heat-related sickness?  If yes, please specify approximately how many days. | ⃝ Yes  ⃝ No  ______________________________days |
| 5. During the last 3 months, do you feel heat has impaired your ability to do any of the following at work? | □ Motivation to complete tasks  □ Speed of task completion  □ Ability to do physical work  □ Focus on task  □ Attention to small details  □ Quality of work  □ Understanding of tasks  □ Maintain your usual productivity |
| 6. How does heat affect other aspects of your work (during the last 3 months)? | □ Absenteeism  □ Feeling tired  □ Feeling sleepy  □ Less productivity  □ Irritation/Interpersonal issues  □ Work related issues with manager  □ Take more time to complete same task  □ Job satisfaction |

**Part 6: Concerning on heat relief measures**

| 1. Which of the following measures have you used to manage heat stress at work, during the last 3 months? | □ Resting in front of a fan  □ Resting in a rest room  □ Resting in an air-conditioned building  □ Using ice packs to cool body  □ Pouring water over head  □ Showering, bathing in cool water  □ Using your own portable fan  □ Drinking slushies/crushed ice/frozen sport drinks  □ Changing clothing during shift  □ Increasing water intake/hydration |
| --- | --- |
| 2. Which of the following measures have you used to manage heat stress outside of work, during the last 3 months? | □ Resting in shade  □ Resting in front of a fan  □ Resting in an air-conditioned room/home  □ Resting in an air-conditioned public space  □ Showering, bath or swimming in cool water  □ Drinking slushies/crushed ice/frozen sports drinks  □ Sleeping for longer, going to bed early, or having naps after work  □ Increasing water intake/hydration after work  □ Increasing water intake/hydration before work  □ Increasing alcohol consumption  □ Decreasing alcohol consumption  □ Exercising to maintain or increase fitness |

Table 1. Comparison of daily dry bulb temperature and WBGT taken from the HOTHAPS program during the actual survey period for cool months and during the time used as a proxy for survey period for cool months

|  | **Cool months-actual**  **(Nov 2021 to Jan 2022)** | | | **Cool months-proxy**  **(Nov 2022 to Jan 2023)** | | | **p-value** |
| --- | --- | --- | --- | --- | --- | --- | --- |
|  | Min | Max | Avg. ± SD | Min | Max | Avg. ± SD |  |
| T_a_ (^o^C) | 24.2 | 29.7 | 27.6 ± 1.2 | 23.5 | 30.3 | 27.3 ± 1.6 | 0.2 |
| WBGT (^o^C) | 20.7 | 27.3 | 24.7 ± 1.4 | 20.0 | 27.3 | 24.5 ± 2.0 | 0.6 |

Table 2. Respondents’ choice of clothing and their perceptions during the cool and hot months

|  | **Cool months,**  **N = 380^1^** | **Hot months,**  **N = 373^1^** | **Difference^2^** | **95% CI^3^** | **p-value** |
| --- | --- | --- | --- | --- | --- |
| **Upper clothes** | | | | | |
| Short sleeves | 21% | 22% | 0.1% | -6%, 6% | > 0.9 |
| Long sleeves | 53% | 78% | 25% | -32%, -18% | < 0.001 |
| T-shirt | 25% | 0% | -25% | 21%, 30% | < 0.001 |
| Other | 0% | 1% | 0.3% | -1%, 1% | > 0.9 |
| **Lower clothes** | | | | | |
| Short skirt | 0% | 0% |  |  |  |
| Long skirt | 1% | 0% | -1% | -0.2%, 2% | 0.13 |
| Long pants | 99% | 99% | 0.3% | -2%, 2% | > 0.9 |
| Other | 0% | 1% | 0.8% | -2%, 0.6% | 0.4 |
| **Layers of outer clothes** | | | | | |
| 1 layer | 44% | 46% | 2% | -9%, 6% | 0.7 |
| 2 layers | 56% | 54% | -2% | -6%, 9% | 0.7 |
| Thick cloth | 0% | 0% |  |  |  |
| Other | 0% | 0% |  |  |  |
| **Perception about outfit material** | | | | | |
| Comfortable | 57% | 75% | 17% | -24%, -10% | < 0.001 |
| Moderately comfortable | 41% | 24% | -17% | 10%, 23% | < 0.001 |
| Uncomfortable | 2% | 1% | -1% | -0.7%, 3% | 0.3 |
| ^1^ % | | | | | |
| ^2^ Hot months – Cool months | | | | | |
| ^3^ CI = Confidence Interval | | | | | |


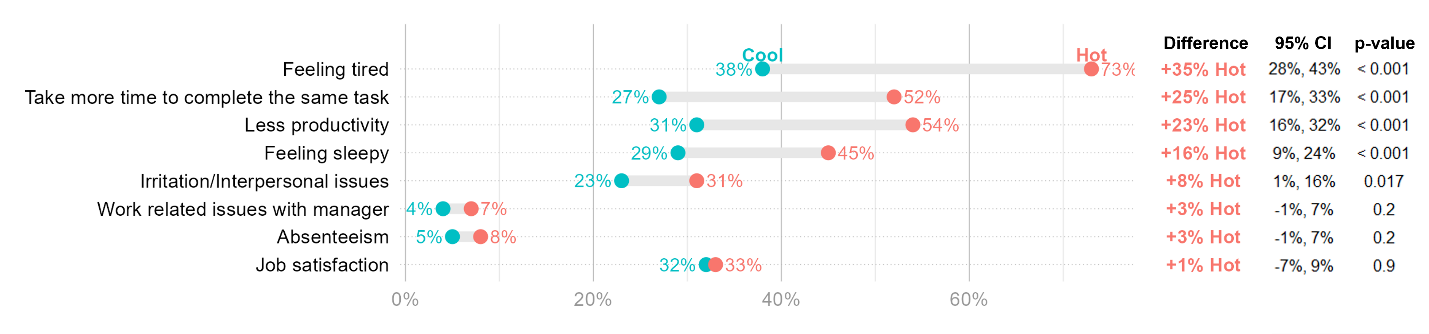


Figure 1. Workers’ perceived impact of heat stress on other aspects of their work during the cool and hot months


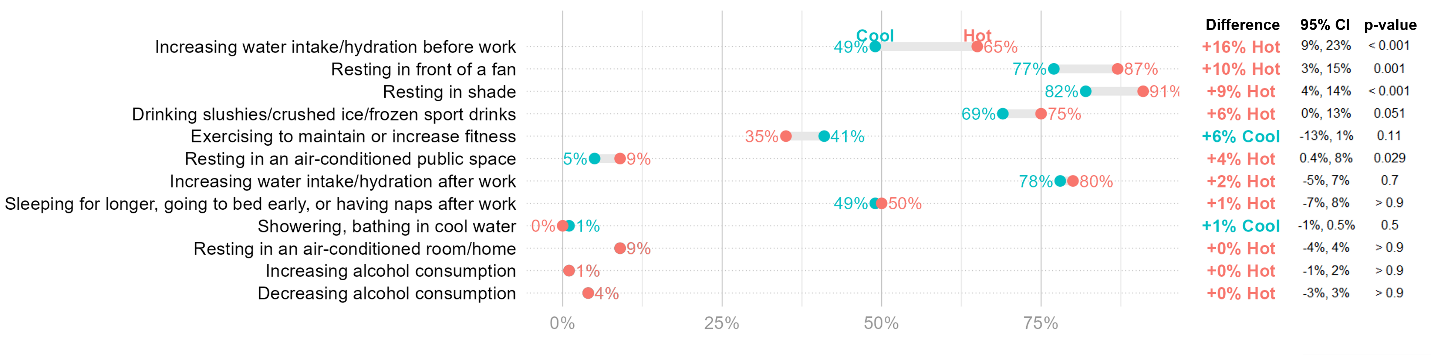


Figure 2. Workers’ heat relief measures outside of their work during the cool and hot months
